# Characteristics of late HIV diagnosis in Germany in 2019 and 2020 - A regional analysis

**DOI:** 10.1007/s15010-026-02935-3

**Published:** 2026-08-25

**Authors:** Carlotta Helbig, Susanne Mohr, Mathias W. Pletz, Juliane Ankert, Jens Forthmann, Markus Bickel, Christoph Stephan, Olaf Degen, Sebastian Noe, Hans Wesselmann, Daniela Tominski, Stefanie Holm, Christoph Spinner, Neel Petersen, Christoph Boesecke, Axel Baumgarten, Wolfgang Schmidt, Sven Becker, Hartmut Stocker, Christoph Lübbert, Thomas Heuchel, Christoph Wyen, Wilfried Obst, Ines Ruck, Steve Rößler, Heribert Knechten, Michael Waizmann, Thomas Seidel, Benjamin T. Schleenvoigt

**Affiliations:** 1 Institute of Infectious Diseases and Infection Control, Jena University Hospital, Friedrich-Schiller-University, Jena, Germany; 2 Department of Psychosomatic Medicine and Psychotherapy, Jena University Hospital, Friedrich-Schiller-University, Jena, Germany; 3 ICH Study Center Hamburg, Hamburg, Germany; 4 Infektiologikum Frankfurt, Frankfurt Am Main, Germany; 5 Department of Internal Medicine, Infectious Diseases, University Hospital Frankfurt, Frankfurt Am Main, Germany; 6 Clinic for Infectious Diseases, University Medical Center Hamburg-Eppendorf, Hamburg, Germany; 7 MVZ München Am Goetheplatz, Munich, Germany; 8 Department of Infectious and Respiratory Medicine, Charité Universitätsmedizin Berlin, Corporate Member of Freie Universität Berlin, Humboldt-Universität Zu Berlin, Berlin, Germany; 9 Department of Infectious Diseases, Vivantes Auguste-Viktoria-Klinikum, Berlin, Germany; 10 Praxis Georgstrasse, Hanover, Germany; 11 Clinical Department of Internal Medicine II, TUM School of Medicine and Health, TUM University Hospital, Munich, Germany; 12 Second Medical Department, University Hospital Schleswig-Holstein, Kiel, Germany; 13 Department of Medicine I, University Hospital Bonn, Bonn, Germany; 14 Center for Infectiology Berlin-Prenzlauer Berg, Berlin, Germany; 15 MVZ Infektiologie Ärzteforum Seestrasse, Berlin, Germany; 16 Diabeteszentrum Erfurt, Erfurt, Germany; 17 Department of Infectious Diseases, St. Joseph Hospital, Berlin-Tempelhof, Berlin, Germany; 18 Division of Infectious Diseases and Tropical Medicine, Department of Medicine I, Leipzig University Medical Center, Leipzig, Germany; 19 Medcenter Chemnitz, Chemnitz, Germany; 20 Praxis Am Ebertplatz, Cologne, Germany; 21 Department I of Internal Medicine, Faculty of Medicine, University Hospital Cologne, Cologne, Germany; 22 Department of Gastroenterology, Hepatology and Infectious Diseases, Otto-Von-Guericke University Magdeburg, Magdeburg, Germany; 23 Praxis Dr. Ruck, Leipzig, Germany; 24 Arztpraxis Rößler, Zwickau, Germany; 25 Laboratory Dr. Knechten, Medical Center for HIV and Hepatitis, Aachen, Germany; 26 Medcenter Leipzig, Leipzig, Germany; 27 Medcenter Weimar, Weimar, Germany

**Keywords:** HIV, PLWH, Late diagnosis, AIDS, CD4

## Abstract

**Purpose:**

A large proportion of HIV diagnoses are made at an advanced stage of infection and different patient characteristics have been associated with late diagnosis of HIV. This study aims to assess the prevalence of late HIV diagnosis, to characterize individuals diagnosed at a late stage of infection and to evaluate regional differences in Germany.

**Methods:**

This retrospective, cross-sectional analysis included 1,169 individuals newly diagnosed with HIV in 2019 and 2020. Data on patient demographics, information on the HIV diagnosis and laboratory parameters were collected. Late diagnosis and advanced HIV disease were defined according to established consensus criteria. Regional comparison was made between eastern and western Germany.

**Results:**

In our cohort, 82.6% of the individuals were male and 16.9% female, with a median age of 39 years (IQR: 31–50). The prevalence of late diagnosis was 61.6% and advanced HIV disease, as a subgroup of late diagnosis, was present in 41.7%. Individuals with late diagnosis were significantly older than those diagnosed at an earlier stage (p < 0.001). Patients in eastern Germany were diagnosed at more advanced stages of infection with significantly higher rates of late diagnosis (p < 0.01), advanced HIV disease (p < 0.001), and AIDS-defining conditions (p < 0.001), as well as significantly lower CD4 cell counts (p < 0.001).

**Conclusion:**

Our data demonstrate that a high proportion of HIV diagnoses in Germany are made at a late stage of infection. Late diagnosis was more frequent in older individuals and in those living in eastern Germany. These findings highlight the need for strengthened HIV testing strategies, particularly in underserved populations and regions.

**Supplementary Information:**

The online version contains supplementary material available at https://doi.org/10.1007/s15010-026-02935-3.

## Introduction

An estimated 97,700 people were living with HIV in Germany in 2024 [1]. UNAIDS has set the global 95–95–95 targets, aiming that 95% of all people living with HIV (PLWH) are diagnosed, 95% of those diagnosed receive antiretroviral therapy (ART), and 95% of those on treatment achieve viral suppression [2]. While Germany has successfully reached the second two targets, the first target remains unmet, with approximately 92% of PLWH aware of their HIV status. A substantial proportion of infections continue to be diagnosed at a late stage, often several years after initial infection, indicating ongoing gaps in early testing and diagnosis [1].

Two terms are commonly used for describing delayed HIV diagnosis (formerly referred to as late presentation). *Late HIV diagnosis* is defined as a diagnosis occurring with a CD4 count below 350 cells/µl or the presence of an AIDS-defining event. *Presentation with advanced HIV disease* is defined as being diagnosed with a CD4 count below 200 cells/µl or an AIDS-defining event [3]. In Germany, approximately one-third of HIV diagnoses occur at a stage of advanced immunodeficiency [1].

Late HIV diagnosis is associated with an increased risk of onward HIV transmission, primarily due to prolonged periods of untreated infection and elevated viral load. In addition, delayed initiation of antiretroviral therapy is linked to poorer treatment outcomes, including higher rates of treatment failure and suboptimal immune recovery [4]. Consequently, individuals diagnosed at a late stage exhibit a higher burden of both infectious and non-infectious comorbidities, including opportunistic infections [5]. Surveillance data on HIV diagnoses in the UK from 2023 showed that people diagnosed late in England were 10 times more likely to die (all-cause mortality) within a year of their diagnosis, compared with people diagnosed early [6].

Several patient characteristics have been associated with late HIV diagnosis. A European study indicated that older patients, heterosexuals and patients from Africa had a higher chance of being diagnosed late [7]. In Germany, Boesecke et al. have found similar patient characteristics (older age, heterosexual transmission and migration background) to be associated with late HIV diagnosis [8]. A regional analysis of data from 2014 showed that individuals in eastern Germany were diagnosed with a higher viral load and with a higher prevalence of AIDS compared to those in western Germany [9]. A Brazilian study found male patients with lower educational level more likely to be diagnosed late [10].

HIV-related stigma has consistently been shown to negatively affect HIV testing behavior, thereby contributing to delayed diagnosis [11]. As a result, stigma and discrimination represent key barriers across the HIV care continuum and significantly impact progress towards the 95–95–95 goals defined by UNAIDS. In addition to these targets, UNAIDS has introduced the objective that less than 10% of people living with HIV should experience stigma and discrimination [2]. Addressing stigma is essential to reduce late HIV diagnoses and improve timely access to testing and care.

To increase the proportion of people aware of their positive HIV status, it is key to understand the clinical circumstances of late HIV diagnosis. This analysis aimed to identify the characteristics of individuals diagnosed late in a large German cohort collaboration, as a prerequisite for improving the achievement of national UNAIDS goals.

## Methods

### General methods

**Fig. 1:**
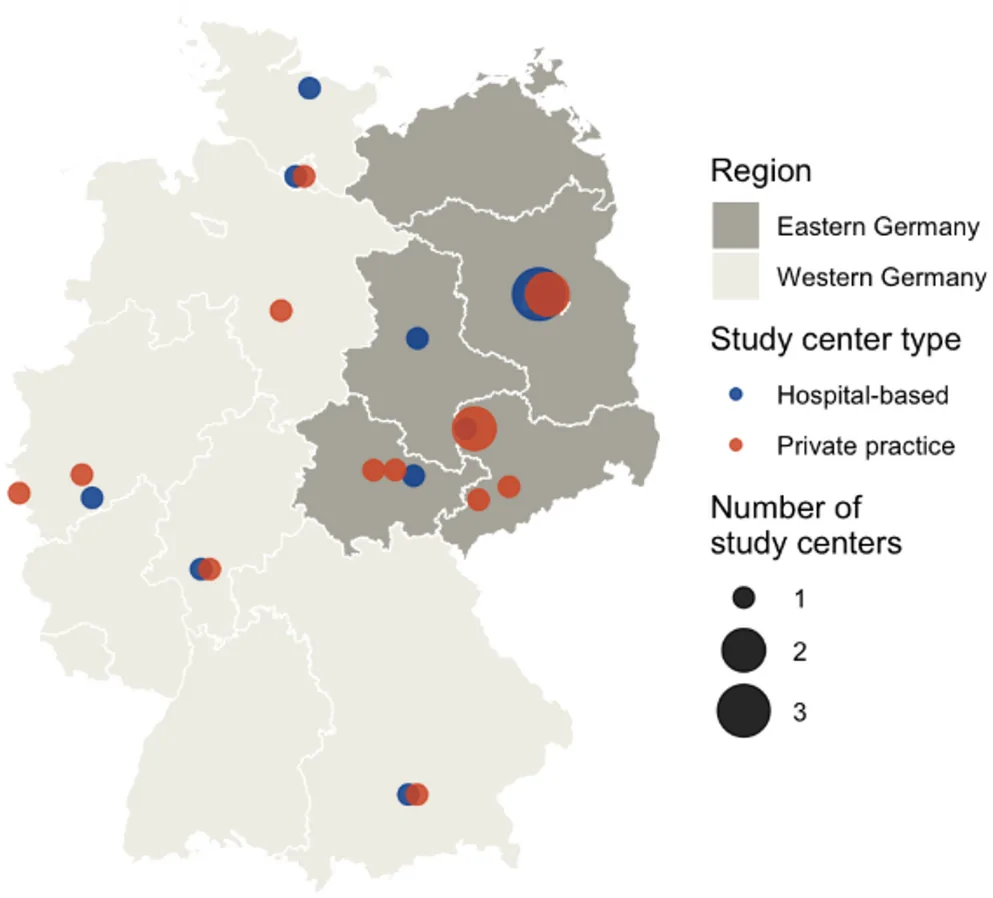
Geographic distribution of the 25 participating study centers across Germany. Marker color indicates study center type (hospital-based outpatient clinic vs. private practice); marker size indicates the number of study centers at a given location. Shaded areas indicate eastern (n = 14 study centers) and western Germany (n = 11 study centers), as defined in Sect. "Regional Comparison"

This is a retrospective, cross-sectional analysis of 1,169 individuals who were newly diagnosed with HIV in 2019 and 2020. Data were collected via decentralized database queries from routine clinical data across 25 study centers, including 11 hospital-based outpatient clinics and 14 private HIV care centers (Fig. 1). Study centers were selected based on prior collaboration in a research network (HIV-regional) with additional centers recruited for this project. Approval from the ethics committee of the University Hospital Jena was obtained (reference number: 4535–08/17).

The dataset included information on patient demographics (gender, birth year, place of residence via postal code), data on the HIV diagnosis (HIV testing site, reason for HIV test, transmission risk behavior, AIDS-defining events) and laboratory data (initial CD4 count and initial viral load). Six individuals identified as diverse gender were excluded from sex-stratified analyses due to the small sample size.

Assessed transmission risk categories included men who have sex with men (MSM), heterosexual contact, intravenous drug use (IVDU), origin from a high prevalence country or pre- or perinatal infection. Up to three risk factors could be recorded per individual, allowing for overlapping exposures.

HIV testing sites were categorized as follows: AIDS support organization (AIDS-Hilfe), blood donation services, central reception centers for asylum seekers, general practitioners, HIV self-testing, hospitals, public health departments, private HIV care centers, and other settings.

Reasons for HIV testing were classified into AIDS-defining event, blood donation screening, HIV-related symptom, patient request, post-exposure testing, pregnancy screening, routine screening during migration/asylum process or other reasons.

Statistical analyses were performed using R version 4.5.1 (The R Foundation for Statistical Computing, Vienna). Categorical variables were described as absolute frequencies and proportions. Continuous variables were reported as median with interquartile range (IQR). Group differences in categorical variables were assessed using the chi-squared test or Fisher's exact test, as appropriate. Continuous variables were compared between groups using the Mann–Whitney *U* test. A p-value of less than 0.05 was considered statistically significant. Missing data were not imputed, proportions were calculated based on available data, with the number of missing values reported where applicable.

### Late diagnosis and advanced HIV disease

*Late HIV diagnosis* was defined as a CD4 cell count below 350 cells/µl at presentation or the presence of an AIDS-defining event, irrespective of CD4 cell count, in accordance with established consensus definitions. *Advanced HIV disease* was defined as a CD4 cell count below 200 cells/µl or an AIDS-defining event at the time of diagnosis, regardless of CD4 cell count [3].

In cases with missing baseline CD4 cell count data, classification was handled as follows: individuals with an AIDS-defining event were categorized as late diagnosed and presenting with advanced HIV disease, while individuals without an AIDS-defining event were classified as “unknown”. For comparative analyses, only individuals classified as late diagnosed or non-late diagnosed were included, the “unknown” group was excluded from these analyses.

### Regional comparison

Regional comparisons were performed between eastern and western Germany. Classification was based on the patients’ postal codes. Western Germany included the federal states Baden-Württemberg, Bavaria, Bremen, Hamburg, Hesse, Lower Saxony, North Rhine–Westphalia, Rhineland-Palatinate, Saarland, and Schleswig–Holstein. Eastern Germany included the federal states Berlin, Brandenburg, Mecklenburg–Western Pomerania, Saxony, Saxony-Anhalt, and Thuringia.

### COVID-19 pandemic

As the study period spans 2019 and 2020 the COVID-19 pandemic may have affected HIV testing and healthcare utilization from March 2020 onwards. We therefore divided the study period into a pre-COVID-19 phase (1 January 2019 to 29 February 2020) and a COVID-19 phase (1 March 2020 to 31 December 2020) according to the onset of the first COVID-19 wave in Germany [12] and compared key parameters between the two phases.

## Results

### Study population characteristics

Data from 1,169 individuals across 25 study centers were included in the analysis. Of these, 966 (82.6%) were male, 197 (16.9%) female, and 6 (0.5%) identified as diverse. The median age of the cohort was 39 years (interquartile range [IQR]: 31–50 years). The most frequently reported transmission risk factor was men who have sex with men (MSM), followed by heterosexual contact (Table 1).

**Table 1:** Study population characteristics

Characteristics	n (%)	male n (%)	female n (%)
Total	1169	966 (82.6%)	197 (16.9%)
*Gender*			
Male	966 (82.6%)	-	-
Female	197 (16.9%)	-	-
Diverse	6 (0.5%)	-	-
*Age*			
Median (IQR)	39 (31–50)	38 (31–50)	40 (31–47)
18–29	206 (17.6%)	171 (17.7%)	35 (17.8%)
30–39	399 (34.1%)	332 (34.4%)	63 (32.0%)
40–49	266 (22.8%)	207 (21.4%)	58 (29.4%)
50–59	211 (18.0%)	181 (18.7%)	29 (14.7%)
60–69	67 (5.7%)	57 (5.9%)	10 (5.1%)
>69	20 (1.7%)	18 (1.9%)	2 (1.0%)
*Transmission risk factor*			
MSM	508 (43.5%)	500 (51.8%)	2 (1.0%)
Heterosexual contact	233 (19.9%)	146 (15.1%)	87 (44.2%)
IVDU	25 (2.1%)	21 (2.2%)	4 (2.0%)
High prevalence country	96 (8.2%)	34 (3.5%)	62 (31.5%)
Pre- or perinatal infection	1 (0.1%)	1 (0.1%)	0
Unknown	345 (29.5%)	294 (30.4%)	51 (25.9%)
*Place of residence (federal state)*			
Baden-Württemberg	5 (0.4%)	5 (0.5%)	0 (0%)
Bavaria	132 (11.3%)	105 (10.9%)	27 (13.7%)
Berlin	182 (15.6%)	153 (15.8%)	29 (14.7%)
Brandenburg	30 (2.6%)	23 (2.4%)	7 (3.6%)
Bremen	2 (0.2%)	1 (0.1%)	1 (0.5%)
Hamburg	165 (14.1%)	135 (14.0%)	24 (12.2%)
Hesse	208 (17.8%)	174 (18.0%)	34 (17.3%)
Lower Saxony	68 (5.8%)	57 (5.9%)	11 (5.6%)
Mecklenburg-Western Pomerania	6 (0.5%)	4 (0.4%)	2 (1.0%)
North Rhine-Westphalia	79 (6.8%)	67 (6.9%)	12 (6.1%)
Rhineland-Palatinate	19 (1.6%)	15 (1.6%)	4 (2.0%)
Saarland	0	0	0
Saxony	89 (7.6%)	74 (7.7%)	15 (7.6%)
Saxony-Anhalt	26 (2.2%)	23 (2.4%)	3 (1.5%)
Schleswig–Holstein	84 (7.2%)	73 (7.6%)	11 (5.6%)
Thuringia	63 (5.4%)	48 (5.0%)	15 (7.6%)
Outside of Germany	11 (0.9%)	9 (0.9%)	2 (1.0%)

### Initial CD4 counts and AIDS-defining events

**Fig. 2:**
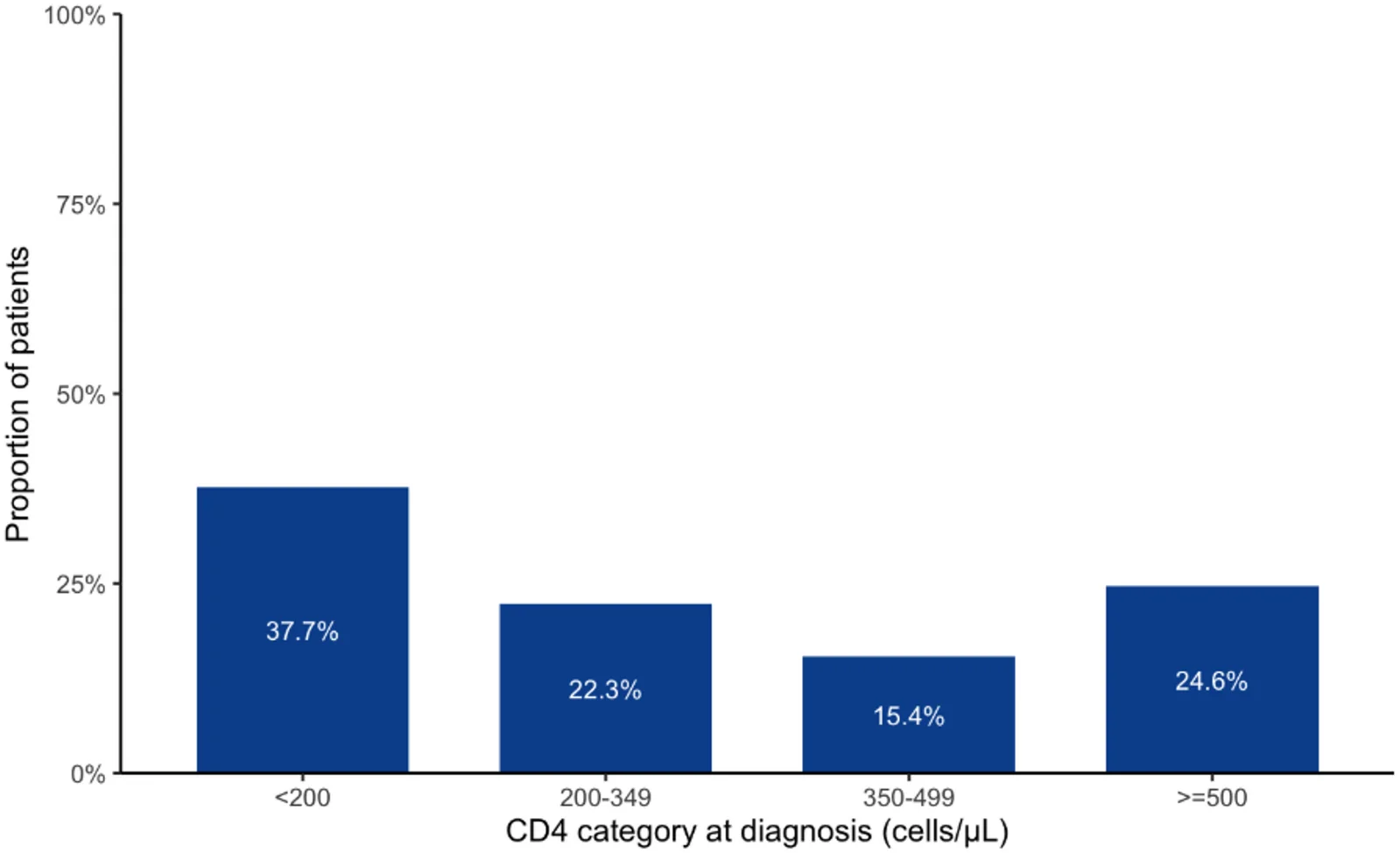
Distribution of CD4 cell count categories at initial HIV diagnosis among 1,119 individuals newly diagnosed with HIV in Germany in 2019 and 2020. CD4 cell count categories are defined as follows: < 200, 200–349, 350–499, ≥ 500 cells/µl. Percentages are based on individuals with available CD4 cell count data; patients with missing baseline CD4 values (n = 50) are not shown

CD4 counts were available for 1,119 (95.7%) patients. The median CD4 count at initial HIV diagnosis was 289 cells/µl with an interquartile range from 108–493 cells/µl. 671/1,119 (60.0%) presented with an initial CD4 count below 350 cells/µl and of these 422/1,119 (in total 37.7%) with an initial CD4 count below 200 cells/µl (Fig. 2). A total of 269 (23.0%) presented with one or more AIDS-defining events at time of their initial HIV diagnosis. The most frequent documented AIDS-defining events were pneumocystis pneumonia (25.2%) followed by candidiasis (19.3%) and wasting (18.3%) (see Supplementary Table 1).

### Prevalence of late diagnosed and presentation with advanced HIV disease

As defined above, 690/1,121 (61.6%) of the patients were classified as late diagnosed and 468/1,121 (41.7%) presented with advanced HIV disease, presenting a subgroup of late diagnosis. 431/1,121 (38.4%) were classified as non-late-diagnosed and 48/1,169 (4.1%) were classified as “unknown” because of non-available data on CD4-counts. 2 patients did not have available CD4 counts but did present with an AIDS-defining event, so they were consequently classified as late diagnosed and presenting with advanced HIV.

#### Late diagnosed – patient characteristics

Overall, 690 (61.6%) of the patients were classified as late diagnosed. The proportion of late diagnosis did not differ significantly in female compared to male patients (65.4% vs. 60.7%, p = 0.4675). People with late diagnosis were significantly older than people who were non-late-diagnosed (Mann–Whitney *U* test, p < 0.001). The median age of the late diagnosed was 42 (IQR: 34–52) and of the non-late-diagnosed 34 (IQR: 29–45). The age group with the highest prevalence of late diagnosis (76.1%) was 60–69 years. Among the transmission risk categories, individuals from high-prevalence countries, people who inject drugs and heterosexuals exhibited the highest prevalence of late diagnosis (74.2%, 65.2%, 60.2% respectively) however, case numbers for these categories were quite small (n = 96, n = 25, n = 233). In particular, the proportion of late diagnosis was significantly higher among individuals with high-prevalence country as a risk factor compared to those without this risk factor (74.2% vs. 60.4%, p = 0.01222).

### HIV test site

**Fig. 3:**
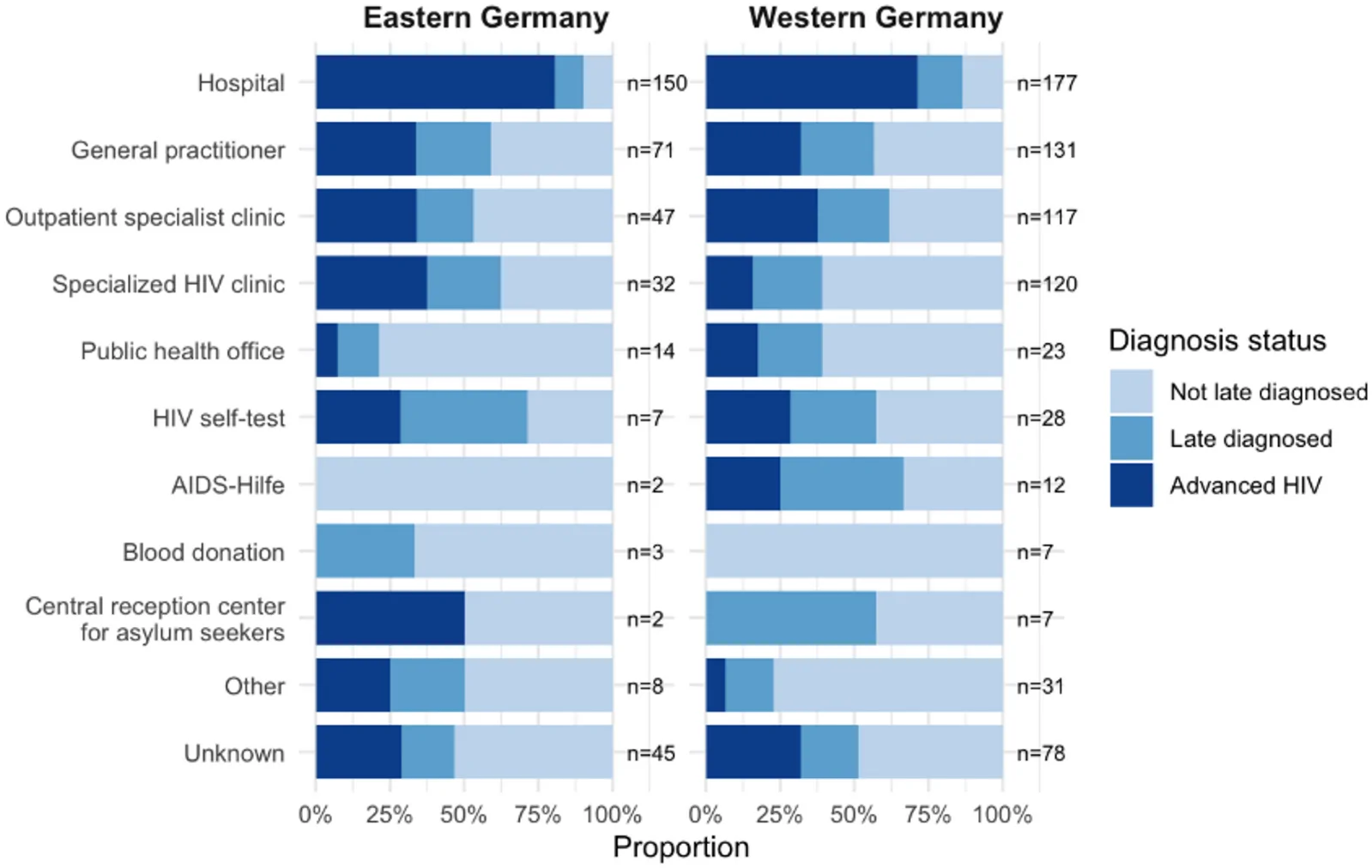
Prevalence of late diagnosis and advanced HIV disease by HIV test site, stratified by region (eastern vs. western Germany) among 1,112 individuals newly diagnosed with HIV in Germany in 2019 and 2020. Late diagnosis was defined as a CD4 cell count below 350 cells/µl or the presence of an AIDS-defining event at the time of diagnosis. Advanced HIV disease, a subset of late diagnosis, was defined as a CD4 cell count below 200 cells/µl or the presence of an AIDS-defining event. Individuals with unknown CD4 cell count and no AIDS-defining event, as well as individuals residing outside of Germany were excluded from this analysis

The most frequent HIV test site was the hospital with 28.7%, followed by general practitioners (17.8%) and outpatient specialist clinics (14.7%). The lowest median CD4 count with 111 cells/µl at initial diagnosis was observed among patients diagnosed in hospital settings. When comparing eastern and western Germany, a higher proportion of individuals were diagnosed in a hospital setting in eastern than in western Germany (38.6% vs. 23.9%) (Table 2). Among hospital-diagnosed individuals, the proportion of late diagnoses was also higher in eastern than in western Germany (90.0% vs. 86.4%) (Fig. 3), however this difference was not statistically significant (p = 0.4132; see Supplementary Table 2 for the corresponding comparison across all test site categories).

**Table 2:** Distribution of HIV test sites, overall and by region (eastern vs. western Germany) with associated CD4 cell counts

HIV test site	Total: n (%)	Eastern Germany: n (%)	Western Germany: n (%)	Median CD4 count at diagnosis [IQR]
Hospital	336 (28.7%)	153 (38.6%)	182 (23.9%)	111 (32–260)
General practitioner	208 (17.8%)	72 (18.2%)	134 (17.6%)	324 (156–493)
Outpatient specialist clinic	172 (14.7%)	50 (12.6%)	122 (16.0%)	305 (136–474)
Specialized HIV clinic	163 (13.9%)	33 (8.3%)	125 (16.4%)	442 (231–637)
Public health office	37 (3.2%)	14 (3.5%)	23 (3.0%)	418 (322–640)
HIV self-test	36 (3.1%)	8 (2.0%)	28 (3.7%)	309 (190–514)
AIDS-Hilfe	14 (1.2%)	2 (0.5%)	12 (1.6%)	346 (254–446)
Blood donation	12 (1.0%)	5 (1.3%)	7 (0.9%)	564 (438–758)
Central reception center for asylum seekers	9 (0.8%)	2 (0.5%)	7 (0.9%)	333 (326–407)
Other	41 (3.5%)	8 (2.0%)	33 (4.3%)	529 (321–657)
Unknown	141 (12.1%)	49 (12.4%)	89 (11.7%)	356 (161–542)

*Regional classification was not applicable for 11 individuals residing outside of Germany; these are included in the total but excluded from the regional columns (n = 1,158 for eastern + western Germany)

**Table 3:** Distribution of reasons for HIV tests with associated CD4 cell counts

Reason for HIV test	Total: n (%)	Eastern Germany: n (%)	Western Germany: n (%)	Median CD4 count at diagnosis [IQR]
HIV-related symptom	365 (31.2%)	162 (40.9%)	201 (26.4%)	228 (100–420)
Patient request	264 (22.6%)	43 (10.9%)	218 (28.6%)	413 (275–607)
AIDS-defining illness	177 (15.1%)	73 (18.4%)	102 (13.4%)	44 (17–110)
Post-exposure testing	64 (5.5%)	13 (3.3%)	48 (6.3%)	426 (214–542)
Pregnancy screening	36 (3.1%)	14 (3.5%)	22 (2.9%)	353 (234–480)
Blood donation screening	12 (1.0%)	5 (1.3%)	7 (0.9%)	564 (438–758)
Routine screening during migration/asylum process	7 (0.6%)	2 (0.5%)	5 (0.7%)	407 (349–642)
Other	78 (6.7%)	29 (7.3%)	49 (6.4%)	376 (201–519)
Unknown	166 (14.2%)	55 (13.9%)	110 (14.4%)	346 (191–505)

*Regional classification was not applicable for 11 individuals residing outside of Germany; these are included in the total but excluded from the regional columns (n = 1,158 for eastern + western Germany)

### Reason for HIV test

**Fig. 4:**
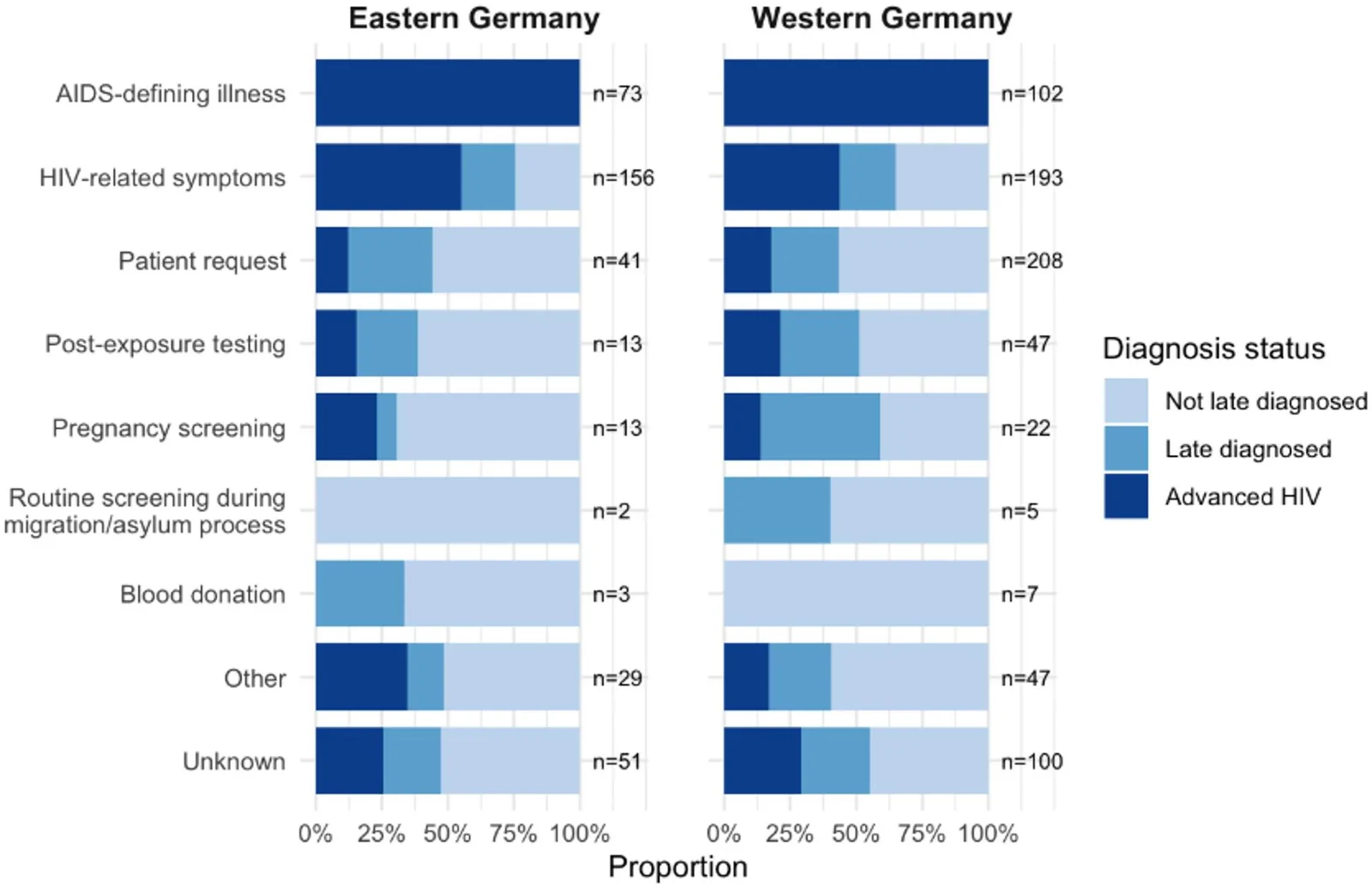
Prevalence of late diagnosis and advanced HIV disease by reason for HIV test, stratified by region (eastern vs. western Germany) among 1,112 individuals newly diagnosed with HIV in Germany in 2019 and 2020. Late diagnosis was defined as a CD4 cell count below 350 cells/µl or the presence of an AIDS-defining event at the time of diagnosis. Advanced HIV disease, a subset of late diagnosis, was defined as a CD4 cell count below 200 cells/µl or the presence of an AIDS-defining event. Individuals with unknown CD4 cell count and no AIDS-defining event, as well as individuals residing outside of Germany were excluded from this analysis

The most common reason for HIV testing was the presence of HIV-related symptoms (31.2%), followed by patient-initiated testing (22.6%) and the presence of an AIDS-defining event (15.1%). The lowest median CD4 cell count at diagnosis was observed among individuals tested due to an AIDS-defining event (44 cells/µl), whereas the highest median CD4 cell count was found among individuals diagnosed through blood donation screening (564 cells/µl) (Table 3, Fig. 4).

**Table 4:** Median CD4 count at diagnosis, prevalence of Late Diagnosis and prevalence of Advanced HIV by federal state

Federal state	n (%)	Median CD4 count at diagnosis [IQR]	Prevalence of Late Diagnosis: n (%)	Prevalence of Advanced HIV: n (%)
Eastern Germany	**396 (34.2%)**	**247 (88–437)**	**257 (67.5%)**	**192 (50.4%)**
Berlin	182 (15.6%)	178 (50–358)	130 (75.6%)	106 (61.6%)
Brandenburg	30 (2.6%)	158 (90–427)	19 (65.5%)	16 (55.2%)
Mecklenburg-Western Pomerania	6 (0.5%)	321 (198–325)	4 (80.0%)	2 (40.0%)
Saxony	89 (7.6%)	336 (162–507)	49 (55.7%)	30 (34.1%)
Saxony-Anhalt	26 (2.2%)	308 (94–373)	15 (57.7%)	11 (42.3%)
Thuringia	63 (5.4%)	280 (112–494)	40 (65.6%)	27 (44.3%)
Western Germany	**762 (65.8%)**	**308 (128–525)**	**430 (58.8%)**	**273 (37.3%)**
Baden-Württemberg	5 (0.4%)	219 (176–360)	3 (75%)	1 (25%)
Bavaria	132 (11.3%)	290 (111–486)	85 (66.9%)	55 (43.3%)
Bremen	2 (0.2%)	452 (246–659)	1 (50%)	1 (50%)
Hamburg	165 (14.1%)	340 (185–552)	81 (52.9%)	43 (28.1%)
Hesse	208 (17.8%)	281 (120–525)	115 (57.2%)	79 (39.3%)
Lower Saxony	68 (5.8%)	264 (106–525)	40 (58.8%)	27 (39.7%)
North Rhine-Westphalia	79 (6.8%)	365 (140–561)	37 (48.7%)	24 (31.6%)
Rhineland-Palatinate	19 (1.6%)	153 (66–578)	12 (63.2%)	11 (57.9%)
Saarland	0	-	-	-
Schleswig–Holstein	84 (7.2%)	277 (111–436)	56 (69.1%)	32 (39.5%)

*Percentages for prevalence of late diagnosis and advanced HIV disease are each calculated among individuals with a known classification for the respective variable; individuals classified as “unknown” (as defined in Sect. "Late diagnosis and advanced HIV disease") were excluded from the corresponding calculation

### Regional comparison

When comparing the patients in eastern Germany to western Germany, the patients in eastern Germany were diagnosed at a more progressed stage of infection compared to those in western Germany. CD4 cell counts were significantly lower (median 247 vs. 308 cells/µl; Mann–Whitney U test, p < 0.001), a higher proportion of patients presented with AIDS-defining conditions (31.8% vs. 18.4%, p < 0.001) and the proportion of individuals classified as late diagnosed was consequently also significantly higher (67.5% vs. 58.8%, p < 0.01), as was the proportion with advanced HIV disease (50.4% vs. 37.3%, p < 0.001) (Table 4).

### COVID-19 pandemic

As stated above, we compared key parameters between the pre-COVID-19 phase (1 January 2019 to 29 February 2020) and the COVID-19 phase (1 March 2020 to 31 December 2020). A total of 790 diagnoses were made during the pre-COVID-19 phase and 379 diagnoses during the COVID-19 phase.

The prevalence of late diagnosis was significantly higher during the COVID phase compared to the pre-COVID-19 phase (67.3% vs. 58.8%, p < 0.01) as was the prevalence of advanced HIV disease (46.9% vs. 39.3%, p < 0.05). The median CD4 cell count at HIV diagnosis was significantly lower during the COVID-19 phase than in the pre-COVID-19 phase (266 vs. 303 cells/µl, p < 0.05, Mann–Whitney *U* test). Similarly, the proportion of individuals presenting with at least one AIDS-defining condition was significantly higher during the COVID-19 phase (27.2% vs. 20.6%, p < 0.01) (Table 5).

**Table 5:** Comparison of key characteristics between the pre-COVID-19-phase and the COVID-19-phase

Characteristic	Pre-COVID-19 phase, n = 790	COVID-19-phase, n = 379	p-value
Median CD4 cell count at HIV diagnosis [IQR]	303 (116–512)	266 (92–452)	p < 0.05
Individuals with at least one AIDS defining condition: n (%)	163 (20.6%)	105 (27.7%)	p < 0.01
Prevalence of late diagnosis: n (%)	443 (58.8%)	247 (67.3%)	p < 0.01
Prevalence of advanced HIV disease: n (%)	296 (39.3%)	172 (46.9%)	p < 0.05

## Discussion

This retrospective cross-sectional analysis aimed to characterize the prevalence of late HIV diagnosis in Germany, to describe patient characteristics in individuals with late HIV diagnosis, and to assess differences between eastern and western Germany. To contextualize our study population, we compared it with national surveillance data from the Robert Koch Institute (RKI), which recorded 3,111 and 2,454 new HIV diagnoses in Germany in 2019 and 2020, respectively [13]. Our cohort of 1,169 individuals represents approximately 21% of the 5,565 HIV diagnoses reported nationally in Germany during the same period (2019–2020). However, as participating study centers were selected based on availability and willingness to participate, the representativeness of our cohort with respect to demographic and regional characteristics warrants closer examination. In our cohort, 82.6% of participants were male, 16.9% female and 0.5% diverse. This was broadly comparable to national RKI surveillance data for 2019 and 2020 combined, where 77.8% of newly diagnosed individuals were male, 22.1% female and 0.1% diverse/non-binary – with a slightly higher proportion of male participants in our study population [13]. The regional distribution of our cohort, however, differed markedly from the national pattern: 34.2% of individuals in our cohort were classified as residing in eastern Germany, compared with only 18.8% of HIV diagnoses reported nationally from the eastern German federal states in 2020 [13]. This substantial overrepresentation of eastern Germany likely reflects the composition of participating study centers and should be considered when interpreting the findings of this study.

### Late diagnosis and advanced HIV disease

Our results demonstrate that a substantial proportion of HIV diagnoses continue to occur at a late stage, including a considerable number of cases presenting with advanced disease. Given the association between advanced immunodeficiency at diagnosis and adverse clinical outcomes, early detection of HIV infection remains a key public health priority, not least to prevent onward transmission.

The overall prevalences of late diagnosis (61.6%), advanced HIV disease (41.7%), and AIDS (23.0%) observed in our cohort were higher than reported in national RKI estimates for 2019 and 2020, where approximately 34% of diagnoses in 2019 and 35% of diagnoses in 2020 were made with advanced immunodeficiency (CD4 < 200 cells/µl or AIDS). Furthermore, 15% and 18%, respectively, presented with AIDS [14, 15]. Differences in data source and methodology between our cohort and national estimates should be considered when interpreting this comparison, as our data are based on documented individual cases. Another German multicenter study (FindHIV), covering the same period (2019–2020), analyzed 706 individuals with HIV first diagnosis and found a prevalence for late diagnosis of 55% and an AIDS prevalence of 20% [16]. In conclusion, it is notable that our population presented with higher rates of late diagnosis, advanced HIV disease and AIDS compared with other data sources.

Our data indicate that individuals in eastern Germany are more likely to be diagnosed with HIV at a late stage compared with those in western Germany. This finding raises the question of whether regional differences in healthcare access, testing practices, or utilization may be contributing to these disparities. In addition, HIV-related stigma may represent an important barrier by reducing both the demand for and the offer of HIV testing, potentially to a greater extent in eastern Germany.

Previous analyses, including data from 2014 that were published by our working group, have shown that individuals newly diagnosed with HIV in eastern Germany were younger, had higher viral loads, and more frequently presented with AIDS-defining events [9]. At that time, approximately 21% of patients presented with at least one AIDS-defining event, whereas this proportion remained similarly high at 23% in our cohort. While earlier data did not demonstrate significant differences in median CD4 cell counts between eastern and western Germany, our findings suggest that such differences may now be emerging [9]. As described above, eastern Germany was overrepresented in our cohort, relative to national diagnosis patterns. Combined with the higher prevalence of late diagnosis observed in this region, this overrepresentation might contribute to the higher overall prevalence of late diagnosis observed in our cohort, compared with other data sources.

In our cohort, the prevalence of late diagnosis did not differ significantly between male and female individuals, though the number of female patients was limited (n=197, 16.9%). However, late diagnosis in women remains a relevant topic across Europe with data from the European Centre for Disease Prevention and Control (ECDC) showing that 54% of newly diagnosed women in the European Region were diagnosed with a CD4 count below 350 cells/µl [17]. Tominski et al. showed that in Germany, women were more likely to present with HIV indicator conditions without being tested, suggesting missed diagnostic opportunities in this group [18].

Our sex-stratified analyses were limited to male and female individuals, as only six patients were documented as diverse gender, precluding meaningful statistical comparison of this group. A further limitation is that transgender status was not recorded as a separate variable in this dataset, meaning that transgender individuals may have been classified as male, female or diverse depending on documentation practices in the study centers. This is a notable limitation, as gender diverse individuals, including transgender and non-binary people, may face distinct barriers to HIV testing and care, potentially resulting in different rates of late diagnosis compared to cisgender individuals. Research specifically addressing HIV late diagnosis in gender diverse populations remains scarce, and future studies with larger sample sizes are needed to adequately capture and address the specific needs of this group. To improve data collection on gender diverse individuals, Tordoff et al. recommend using a validated two-step question to separately capture a respondent’s current gender identity and sex assigned at birth [19]. As this approach relies on patient self-report, it is most readily applicable to prospective studies, however, it should also be implemented at the level of patient records and national surveillance systems, so that such data become available for future retrospective analyses.

Furthermore, late diagnosed individuals were significantly older than those diagnosed at an earlier stage, consistent with findings from earlier studies [7, 8, 20]. This association is likely to be caused by different reasons: on the patient side, older individuals have been reported to show lower self-perceived HIV risk and more limited knowledge of HIV and transmission routes [21]. On the healthcare provider side, this may reflect lower clinical suspicion of HIV infection in older individuals, potentially due to assumptions about reduced sexual activity or perceived lower risk [22]. However, increasing life expectancy, prolonged sexual health, and continued sexual activity into older age in the general population underscore the need to consider HIV testing across all age groups [23]. Especially because older individuals tend to have more frequent healthcare contacts, this could offer multiple opportunities for HIV-testing offers and potentially earlier diagnosis [24].

These findings highlight the need to increase awareness among healthcare providers and to expand routine and indicator condition-guided HIV testing, particularly in populations that may not traditionally be perceived as high risk.

### HIV test site and reason for HIV test

Most HIV diagnoses in this cohort were made in hospital settings. This finding could reflect the high proportion of late diagnoses and presentations with AIDS-defining conditions, which often require inpatient care. At the same time, it may indicate missed opportunities for earlier diagnosis in other healthcare settings, such as primary care or outpatient specialist services. As no data was collected on healthcare contacts prior to the HIV diagnosis, this assumption cannot be confirmed within the present study. However, in Finland, 42% of people diagnosed with HIV between 2011 and 2019 had visited a primary care physician between HIV acquisition and diagnosis, and among those diagnosed with a CD4 count below 200 cells/µl, 58% had had primary care contact during this period [25].

The most common reason for HIV testing in our cohort was the presence of HIV-related symptoms. A limitation of this analysis is the lack of standardized data on the specific symptoms leading to testing. Future prospective studies should therefore include more detailed and structured documentation of symptoms or HIV indicating conditions at time of HIV testing. Improving awareness of HIV-associated indicator conditions among healthcare providers may further facilitate earlier testing and diagnosis. Specific interventions in primary care could help to reduce missed opportunities for earlier HIV testing in the German healthcare context. For example, a randomized controlled trial in primary healthcare centers in Catalonia, Spain demonstrated that automated electronic health record reminders triggered by the diagnosis of an HIV indicator condition significantly increased HIV testing rates [26]. Implementing similar electronic prompts within German primary care information systems could support earlier testing among patients presenting with indicator conditions.

Offering HIV testing to individuals presenting with indicator conditions constitutes a risk-based testing approach. As such, some HIV diagnoses might be missed, and this approach remains susceptible to bias and stigma – for example, older individuals may be tested less frequently due to assumptions about their risk. A screening-based approach could therefore represent an alternative strategy to diagnose more individuals, particularly at an earlier, asymptomatic stage of infection. Consequently, a further structural barrier to earlier HIV diagnosis is the legal requirement for informed consent prior to testing (an opt-in approach) in Germany. This requirement originated in the 1980s and 1990s, reflecting concerns about stigma, discrimination, and the “right not to know” at a time when HIV carried a poor prognosis [27]. Given the substantial improvements in HIV treatment and life expectancy since then, this opt-in approach may today constitute an unintended barrier to routine HIV screening, as pre-test counseling requires additional time and resources. This distinct legal treatment of HIV testing, compared with most other diagnostic procedures, may itself contribute to perceptions of HIV as exceptional and thereby reinforce stigma. European and global guidelines already no longer recommend written consent as a precondition for HIV testing; nonetheless, a recent survey by Stengaard et al. found that one third of European countries, including Germany, were still not adhering to World Health Organization (WHO) and European Centre for Disease Prevention and Control (ECDC) recommendations in this regard [28]. In the UK, an opt-out approach has been established in more than 80 emergency departments [29]. In 2023, 95% of people living with HIV in the UK were aware of their diagnosis, compared with 92% in Germany; the UK has thus already met the UNAIDS 95–95-95 targets, a goal Germany has yet to achieve [30, 31]. While Germany continues to rely on an opt-in approach, evidence remains limited: a study from an emergency department in Berlin, Germany using an opt-in screening program, found that among 2,406 individuals tested for blood borne viruses, 1.5% tested positive for HIV with a median CD4 cell count of 213 cells/µl [32]. Although this single-center setting is not representative of Germany as a whole, it suggests that screening-based approaches deserve broader consideration in this context. In line with current international guidelines, we recommend that Germany reconsiders its opt-in written consent requirement for HIV testing and moves towards an opt-out approach, which could help normalize and destigmatize HIV testing.

When comparing HIV test sites between eastern and western Germany, regional differences were observed: 38.6% of diagnoses in eastern Germany were made in a hospital setting, compared with 23.9% in western Germany. However, it remains unclear how this difference relates to the higher proportion of late diagnoses in eastern Germany. On the one hand, it may simply reflect this pattern, since hospitals naturally have a higher prevalence of late and advanced HIV disease, as acutely symptomatic individuals are more likely to be admitted and diagnosed there. On the other hand, it is also possible that both the composition of participating study centers, which included a comparatively high number of hospital-based outpatient clinics, and a distinct HIV care landscape, may have contributed to the observed different prevalence of late diagnosis—regionally and overall. Consequently, the distribution of participating study center types represents an important limitation of this study. In eastern Germany, 6 hospital-based outpatient clinics and 8 private clinics were included, compared with 5 and 6, respectively, in western Germany. The recorded test site does not necessarily correspond to the study center type – for instance, a patient diagnosed in hospital may later have been documented at a private practice. However, individuals may have been more likely to be captured by the study center at which they were also diagnosed. The uneven distribution of study centers may therefore have influenced the representativeness of the observed test site distribution, and consequently, the higher prevalence of late diagnosis in eastern Germany.

Taken together, our cohort’s representativeness of the national HIV epidemic in Germany may be limited, due to the non-random selection and uneven regional distribution of participating study centers. Future studies should aim for a more balanced sampling of study centers across regions and center types to allow for more robust regional comparisons. In addition, national surveillance data could be systematically stratified by region with respect to the prevalence of late diagnosis to robustly compare eastern and western Germany.

The observations in our study highlight the need to strengthen HIV testing strategies and improve awareness and access to care in order to facilitate earlier diagnosis and reduce regional disparities. In line with the UNAIDS objectives on stigma and discrimination, future studies should further investigate whether regional differences in HIV-related stigma are associated with the proportion of late diagnoses among newly diagnosed individuals with HIV.

### COVID-19 pandemic

Our data suggest that diagnoses were made at a later stage of infection during the COVID-19 pandemic in Germany, as reflected by higher rates of late diagnosis and advanced HIV disease, lower CD4 cell count at first diagnosis, and a higher proportion of AIDS-defining conditions in the COVID-19 phase. This likely reflects reduced access to and uptake of HIV testing during the pandemic. National surveillance data of the Robert-Koch-Institute (RKI) for 2020 may provide additional context: although the overall number of new HIV diagnoses declined slightly, the absolute number of late diagnoses remained stable, resulting in a small proportional increase of late HIV diagnoses [14].

These findings are consistent with a single-center retrospective study from Bonn, Germany, which compared Late Diagnosis rates before and during the COVID-19 pandemic (1 January 2019 to 1 October 2021) and reported an increase in late diagnosis from 59% in the pre-COVID-19 period to 83% during the pandemic, with both a CD4 count below 350 cells/µl and below 200 cells/µl being significantly associated with the COVID-19 pandemic [33]. While our results show a similar trend, the effect was less pronounced, which may partly be explained by the limited follow-up of our study. As our study period ended in December 2020 it only captures the first wave of the pandemic and may therefore underestimate the full impact. This is particularly relevant given the natural course of HIV infection, where progression from primary infection to advanced immunodeficiency or AIDS can span several years [34], meaning that reduced testing during later phases of the pandemic may not yet be reflected in our data. However, these findings highlight the need for resilient HIV testing strategies and continued public awareness efforts, especially in the context of future possible pandemics.

## Conclusion

A substantial proportion of HIV diagnoses in Germany continues to occur at a late stage of disease. Late diagnosis was more frequently observed in older individuals, highlighting potential gaps in risk perception and testing practices within this group. In addition, individuals in eastern Germany were more likely to be diagnosed late, possibly pointing to persistent regional disparities, although the overrepresentation of eastern Germany in our cohort suggests that this finding should be interpreted with caution and warrants further investigation in more representative samples.

Taken together, these findings suggest that important opportunities for earlier HIV diagnosis are still being missed. Future studies should aim for a representative sampling of study centers and demographic characteristics and should mandate the coding of HIV indicator conditions at the time of testing. Alongside methodological improvements, strengthening targeted and routine HIV testing strategies, particularly in underserved populations and regions, will be essential to improve early diagnosis and to work towards the UNAIDS 95–95–95 targets and especially the broader goal of reducing HIV-related stigma and discrimination.

## Supplementary Information

Below is the link to the electronic supplementary material.

Supplementary file1 (DOCX 16 kb)

## Data Availability

The data are stored electronically at Jena University Hospital and are available upon reasonable request.
