# Supplementary material for: Characteristics of late HIV diagnosis in Germany in 2019 and 2020 - A regional analysis
Source: Infection. 2026 Aug 25;54(5):2881–94. doi: 10.1007/s15010-026-02935-3 (PMC13727839; doi:10.1007/s15010-026-02935-3)
Supplement: Supplementary file 1 [file 15010_2026_2935_MOESM1_ESM.docx]

**Appendix**

***Supplementary Table 1: Distribution of AIDS-defining events at initial HIV diagnosis and associated CD4 cell counts***

| **AIDS-defining event** | **n (%)** | **Median CD4 count at diagnosis [IQR]** |
| --- | --- | --- |
| Pneumocystis pneumonia | 102 (25.2%) | 34 (15-61) |
| Candidiasis | 78 (19.3%) | 42 (22-106) |
| Wasting | 74 (18.3%) | 63 (24-149) |
| CMV infection | 37 (9.2%) | 28 (13-74) |
| Kaposi sarcoma | 24 (5.9%) | 50 (18-151) |
| HIV encephalopathy | 21 (5.2%) | 65 (38-114) |
| Cerebral toxoplasmosis | 20 (5%) | 54 (31-110) |
| Tuberculosis | 15 (3.7%) | 73 (44-222) |
| Lymphoma | 13 (3.2%) | 98 (57-149) |
| PML | 4 (1.0%) | 119 (52-198) |
| Atypical Mycobacteriosis | 3 (0.7%) | 6 (4-23) |
| Recurrent pneumonia | 3 (0.7%) | 40 (26-363) |
| Others | 10 (2.5%) | 40 (28-72) |

***Supplementary Table 2: Proportion of late diagnosis and advanced HIV disease by HIV test site and region (eastern vs. western Germany)***

| **HIV test site** | **Late diagnosis** | | **Advanced HIV** | |
| --- | --- | --- | --- | --- |
|  | **Eastern Germany** | **Western Germany** | **Eastern Germany** | **Western Germany** |
| Hospital | 135/150 (90.0%) | 153/177 (86.4%) | 121/150 (80.7%) | 126/177 (71.2%) |
| General practitioner | 42/71 (59.2%) | 74/131 (56.5%) | 24/71 (33.8%) | 42/131 (32.1%) |
| Outpatient specialist clinic | 25/47 (53.2%) | 72/117 (61.5%) | 16/47 (34.0%) | 44/117 (37.6%) |
| Specialized HIV clinic | 20/32 (62.5%) | 47/120 (39.2%) | 12/32 (37.5%) | 19/120 (15.8%) |
| Public health office | 3/14 (21.4%) | 9/23 (39.1%) | 1/14 (7.1%) | 4/23 (17.4%) |
| HIV self-test | 5/7 (71.4%) | 16/28 (57.1%) | 2/7 (28.6%) | 8/28 (28.6%) |
| Blood donation | 1/3 (33.3%) | 0/7 (0.0%) | 0/3 (0.0%) | 0/7 (0.0%) |
| Central reception center for asylum seekers | 1/2 (50.0%) | 4/7 (57.1%) | 1/2 (50.0%) | 0/7 (0.0%) |
| Other | 4/8 (50.0%) | 7/31 (22.6%) | 2/8 (25.0%) | 2/31 (6.5%) |
| Unknown | 21/45 (46.7%) | 40/78 (51.3%) | 13/45 (28.9%) | 25/78 (32.1%) |
